# Diseases Admitted under the Care of the Physicians of the Westminster Hospital, from the 18th of March to the 18th of April, 1800

**Published:** 1800-05

**Authors:** 


*Diſeaſes admitted under the Care of the Phyſicians of the Weſtminſter Hoſpital, from the* 18*th of March to the* 18*th of April,* 1800.

| | | | |
|---|---|---|---|
| Continued Fever | 9 | Aſthenia | 3 |
| Pleuriſy | 1 | Aſthma | 1 |
| Eryſipelas | 1 | Catarrh | 3 |
| Sore Throat | 1 | Chorea | 1 |
| Meaſles | 2 | Convulſions | 2 |
| Amenorrhœa | 4 | Cough | 14 |
| Anaſarca | 4 | Diarrhœa | 4 |
| Aphthæ | 1 | Dyſentery | 1 |
| Aſcites | 3 | Dyſpepſia | 1 |

Dyſuria

| | | | |
|---|---|---|---|
| Dysuria - - - - - | 1 | Menorrhagia - - | 2 |
| Enterodynia - - | 1 | Paralysis - - - | 3 |
| Gastrodynia - - | 1 | Phthisis - - | 4 |
| Hooping Cough - | 3 | Prurigo - - - | 1 |
| Hydrocephalus - - | 1 | Rheumatism - - | 7 |
| Impetigo - - - | 3 | Struma - - - | 1 |
| Itch - - - - - | 2 | Tabes - - - | 2 |
| Jaundice - - - | 2 | Vertigo - - - | 1 |
| Leucorrhœa - - - | 1 | Vomiting - - | 2 |
| Lumbago - - - | 2 | Worms - - - | 1 |

